# Impact of the “Flavescence Dorée” Phytoplasma on Xylem Growth and Anatomical Characteristics in Trunks of ‘Chardonnay’ Grapevines (*Vitis vinifera*)

**DOI:** 10.3390/biology11070978

**Published:** 2022-06-28

**Authors:** Attilio Rizzoli, Luca Jelmini, Gianni Boris Pezzatti, Mauro Jermini, Olivier Schumpp, Christophe Debonneville, Enrico Marcolin, Patrik Krebs, Marco Conedera

**Affiliations:** 1 Agroscope, Campus di Ricerca, a Ramél 18, CH-6593 Cadenazzo, Switzerland; luca.jelmini@gmail.com (L.J.); mauro.jermini@agroscope.admin.ch (M.J.); 2 Swiss Federal Research Institute WSL, Insubric Ecosystem Research Group, Campus di Ricerca, a Ramél 18, CH-6593 Cadenazzo, Switzerland; boris.pezzatti@wsl.ch (G.B.P.); patrik.krebs@wsl.ch (P.K.); marco.conedera@wsl.ch (M.C.); 3 Agroscope, Route de Duillier 50, P.O. Box 1012, CH-1260 Nyon 1, Switzerland; olivier.schumpp@agroscope.admin.ch (O.S.); christophe.debonneville@agroscope.admin.ch (C.D.); 4 TESAF Department, University of Padova, Viale dell’Università 16, I-35020 Legnaro, PD, Italy; enrico.marcolin@unipd.it

**Keywords:** dendroecology, drought-stress, grapevine yellows, SPEI, southern Switzerland

## Abstract

**Simple Summary:**

Annual rings of the susceptible grapevine cultivar ‘Chardonnay’ were measured and used in order to analyse the impact of the Flavescence dorée (FD) infection on the growth in diameter and the anatomical structure of grapevine trunks. Grapevines are susceptible to water shortage and reduce their growth in diameter in the case of summer drought. However, in the case of the expression of FD symptoms, the ring width reductions are extreme and supersede the drought-induced effects. In addition, in coincidence of the FD symptomatic expression, the anatomy of the phloem tissue of infected grapevines appears heavily disarranged. Moreover, sometimes also the formation of the woody ring is incomplete (early wood only). In conclusion, even though the FD phytoplasma does not inhabit and replicate inside the xylem tissue, our results confirm existing indirect inhibiting effects on the ring growth and the xylem tissue formation in FDp-infected grapevines.

**Abstract:**

Flavescence dorée (FD) is a grapevine disease caused by ‘*Candidatus* Phytoplasma vitis’ (FDp), which is epidemically transmitted by the Nearctic leafhopper *Scaphoideus titanus*. In this study, we applied dendrochronological techniques to analyse the response to FDp infections in terms of wood ring widths and anatomical structures of the xylem and phloem tissues of the trunk of the susceptible grapevine cultivar ‘Chardonnay.’ As a rule, grapevines are susceptible to water shortage and reduce their growth in diameter in case of summer drought. In the season of the external expression of FD symptoms, however, the ring width reductions are extreme and supersede any drought-induced effects. In addition, the anatomy of the phloem tissue in the year of the FD symptom expression appears heavily disarranged. Moreover, in the most suffering individuals, the xylem formation remains incomplete and mostly limited to the early wood tissue. In conclusion, even though the FD phytoplasma does not inhabit and replicate inside the xylem tissue, our results confirm existing indirect inhibiting effects on the ring growth and the xylem tissue formation in FDp-infected grapevines.

## 1. Introduction

Flavescence dorée (FD) is a grapevine disease caused by ‘*Candidatus* Phytoplasma vitis’ (FDp) [1,2], which results in severe damage or even death to grapevines (*Vitis* spp.) with detrimental agronomic consequences [3]. FDp is a quarantine organism in Europe and its epidemic transmission is assured by the highly competent Nearctic leafhopper *Scaphoideus titanus* [4,5,6].

Phytoplasmas are usually confined in the sieve elements from where they release defence and stress-related effectors that may spread through the vascular system and affect the distal plant organs and tissues, altering their physiology and structures [7,8,9]. As a consequence, the phytoplasma-induced impairment of the host plants is usually associated with non-specific symptoms, such as chlorosis, premature reddening, leaf curl, abnormal growth, or reduced vigour, and may highly vary according to the concerned host-pathogen combination [10]. On grapevines, external visible FDp-induced symptoms mainly consist of colour alterations of the leaf surface and veins, with a yellowing (white-fruited cvs) or reddening (red-fruited cvs) of the lamina. Leaf margins tend to roll downwards, forming an arrow-like shape and with time, discoloured areas necrotize and dry out. According to the timing of symptom expression, shoots remain thin, rubbery, and unlignified, with a reduced fruit setting (early infections) or just carry brown and shrivelled bunches with dry peduncles (late infections). At the end of the vegetation season, the affected shoots tend to become brittle, and the parts that did not lignify turn black and die [6,11,12]. Recently, a detailed investigation of leaf midribs of FDp-infected grapevines revealed anomalies at the ultrastructural level, with lipids accumulation in the chloroplasts of parenchyma cells [13], as well as a Ca^2+^ influx into the sieve tubes, which may lead to callose deposition, protein plugging, and, eventually, to the occlusion of the sieve plates [14]. Furthermore, indirect consequences of FDp infections have also been reported on the phloematic and xylematic structures of the vines [11,15]. In particular, Jelmini et al. [16] demonstrated that the stems of FDp-infected shoots show hyperplasia of phloem tissues, an irregular arrangement, or a complete lack of fibre-sclereids in the axial phloem, and a general reduction in xylem formation. Although the expression level of such anomalies may significantly differ among cultivars according to their susceptibility to FDp, the infected stems generally fail to normally develop and lack the formation of a periderm. Furthermore, Jelmini et al. [16] found the first stages of anatomical modifications also in the stems of shoots without external FDp symptom expression in FDp-infected grapevines of the susceptible cultivar ‘Chardonnay.’ On the other hand, less susceptible cultivars may show a spontaneous disappearance of external symptoms and recover, although not achieving any kind of resistance to subsequent infections [2,17].

Expanding and deepening the knowledge about possible impacts of an FDp infection on xylem and phloem tissues within grapevines may, thus, help to better understand the epidemic evolution at the individual plant level, including possible interactions with additional environmental (i.e., drought) or pathogenic-induced stress factors [18].

In this respect, the annual xylem growth rate (i.e., the wood rings of the trunk) may be used as a general vitality and resilience indicator to environmental stresses, such as drought or pathogens in woody plants [19,20]. In this study, we applied dendrochronological techniques to the wood rings and analysed the anatomical structures of the phloem and xylem tissues of the trunk of the susceptible grapevine cultivar ‘Chardonnay’ in order to verify their response to FDp infections. We aimed, in particular, at (i) assessing the impact of the emergence of FDp external symptoms on the xylem growth of the year at the trunk level; (ii) detecting existing anatomical alterations of the phloem and xylem tissues in the trunks of FDp-infected specimens; and (iii) identifying possible synergetic effects with environmental stressors, such as summer drought years or site conditions.

## 2. Materials and Methods

### 2.1. Study Area and Sampling Design

**Figure 1:**
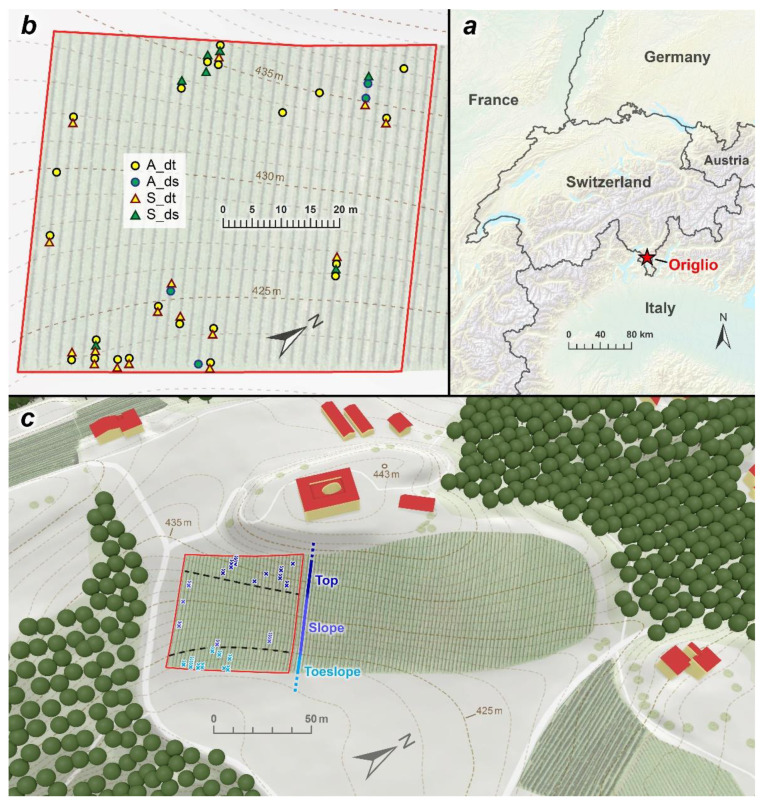
Study area and distribution of the sampled grapevines. (**a**) location of the vineyard; (**b**) detailed map of the sampled individuals; (**c**) geomorphological slope gradient within the vineyard. A_dt = Asymptomatic and drought-tolerant; A_ds = Asymptomatic and drought-sensitive; S_dt = Symptomatic and drought-tolerant; S_ds = Symptomatic and drought-sensitive.

The study area is represented by a vineyard located in Origlio (WGS84 46.043, 8.941; 430 m a.s.l.; Canton Ticino; Southern Switzerland, Figure 1a,b) along a gentle slope (15% on average), spanning from the top of a hill to the toeslope. The climate is Insubric, with a mean annual temperature of 12.4 °C and mean annual precipitation of 1559 mm (climatic normal 1981–2010, meteorological station of Lugano [21]).

The vineyard consists of 2166 roughly coeval (i.e., 16 years old) grapevines, of the FD-susceptible *Vitis vinifera* cv. ‘Chardonnay’ [22], distributed over 34 rows and planted parallel to the steepest descent of the hill (i.e., not arranged on terracing), covering a total area of 3550 m^2^. The grapevines have always been trained with the simple Guyot pruning system, allowing for the exclusion of differences in the diametric growth rate of the trunks due to differing training systems. FD-related symptoms were first observed in 2006 and have been regularly detected every year since then by the local Plant Protection Service despite regular insecticide treatments against the main FDp vector, *S. titanus*, and the systematic rogueing of infected grapevines.

The vineyard was divided into three sectors along an assumed gradient of possible water shortage that may endure during drought years: hilltop, slope, and toeslope (Figure 1c). During the 2019 vegetative season, for each slope sector, all originally planted (i.e., 16 years old) grapevines, developing for the first time typical external symptoms of grapevine yellows (GY), were marked, and the leaves were collected to assess the presence of FDp through qPCR [23]. The same procedure was conducted on a corresponding number of nearby-located asymptomatic and apparently healthy coeval grapevines, i.e., specimens without any external symptoms related, not only to GY but also to any viruses or fungal infections [24]. Leaf sampling and subsequent molecular analyses were conducted between July and September before the trunk coring.

### 2.2. Nucleic Acid Extraction

Petioles and midribs from 3–4 different leaves per specimen equivalent to 0.5 to 1 g were ground in 6 mL of CTAB buffer (3% CTAB, 1.4 M NaCl, 25 mM EDTA, 1 M Tris, pH 8.0) using a Homex grinder (Bioreba). Two mL of homogenate was centrifuged for 10 min at 1000× *g*. 900 μL of supernatant were mixed with 2 μL of β-Mercaptoethanol and shaken for 30 min at 600 rpm and at 65 °C. Chloroform/Isoamylalcohol (900 μL) were added, homogenized by vortexing for 5 s, and centrifuged for 5 min at 3000× *g*. The aqueous layer was carefully transferred to a new tube, mixed with an equal volume of cold isopropanol, and incubated for 30 min at −20 °C for DNA precipitation. Precipitated material was recovered by 2 min of centrifugation at 10,000× *g* and washed with 1 mL of 70% Ethanol. The DNA pellets were dried overnight at room temperature and resuspended into 100 μL of PCR-grade water.

### 2.3. FDp Detection

The FDp status of each sample was determined by triplex qPCR, including a *Vitis* host gene as the extraction control, according to Pelletier et al. [23], using the GoTaq Probe qPCR kit (Promega). The presence of “Bois Noir” phytoplasma (BNp) was also tested since BNp causes the same set of external symptoms on grapevines as FDp and is only distinguishable through PCR analysis [3]. Cycling conditions were 5 min at 95 °C followed by 42 cycles of 15 sec at 95 °C and 30 sec at 60 °C, using a CFX96 real-time PCR instrument (Bio-Rad).

Samples with a Cq value below the limit of repeatability (LR) were considered positive (FDp+). LR is determined using dilution series. It is set at the lowest concentration of the pathogen that can consistently be detected in the grapevine matrix using at least six technical replicates [25].

Samples positive to BNp or of dubious status, i.e., with Cq values above the LR, were excluded from further analyses.

### 2.4. Core Samples’ Collection and Preparation

Based on the results of the molecular analysis, 28 FDp-infected grapevines (FDp+), and a corresponding number of asymptomatic and FDp-free specimens (FDp-) were cored in October 2019 using a common hole-puncher usually used for leather work (0.5 cm punching diameter), which provides results similar to a Trephor micro-corer [26]. The coring point on the trunk was set to an intermediate height between the grafting point and the crown insertion. The resulting cores (0.5 cm in diameter and 1.5 cm long) were stored in distilled water until further manipulation in the laboratory.

For each sampled specimen, a thin section of 15 µm was prepared using the WSL-Lab-Microtome [27], stained with safranin (0.8% *w*/*v*) and astrablue (0.5% *w*/*v*) in order to highlight the tissues containing lignin and cellulose, respectively, and finally fixed on glass slides with Eukitt [27].

### 2.5. Image Capturing and Processing

For each obtained thin section, high-resolution (0.678 px/µm) digital images were taken by microscope (Olympus BX53 with UPlanApo objective, magnitude 5×, Olympus Corporation, Tokyo, Japan) using the software cellSens (version 1.16, Olympus Corporation). Each image was quality checked, and incomplete (e.g., missing phloem or external xylem rings) or broken samples (e.g., cracks within the annual rings) were discarded from further processing. After this process, the final dataset consisted of 23 symptomatic (and FDp-infected) and 26 asymptomatic (and FDp-free) samples (Figure 1b).

### 2.6. Data Analysis

#### 2.6.1. Assessment of Anatomical Anomalies

**Figure 2:**
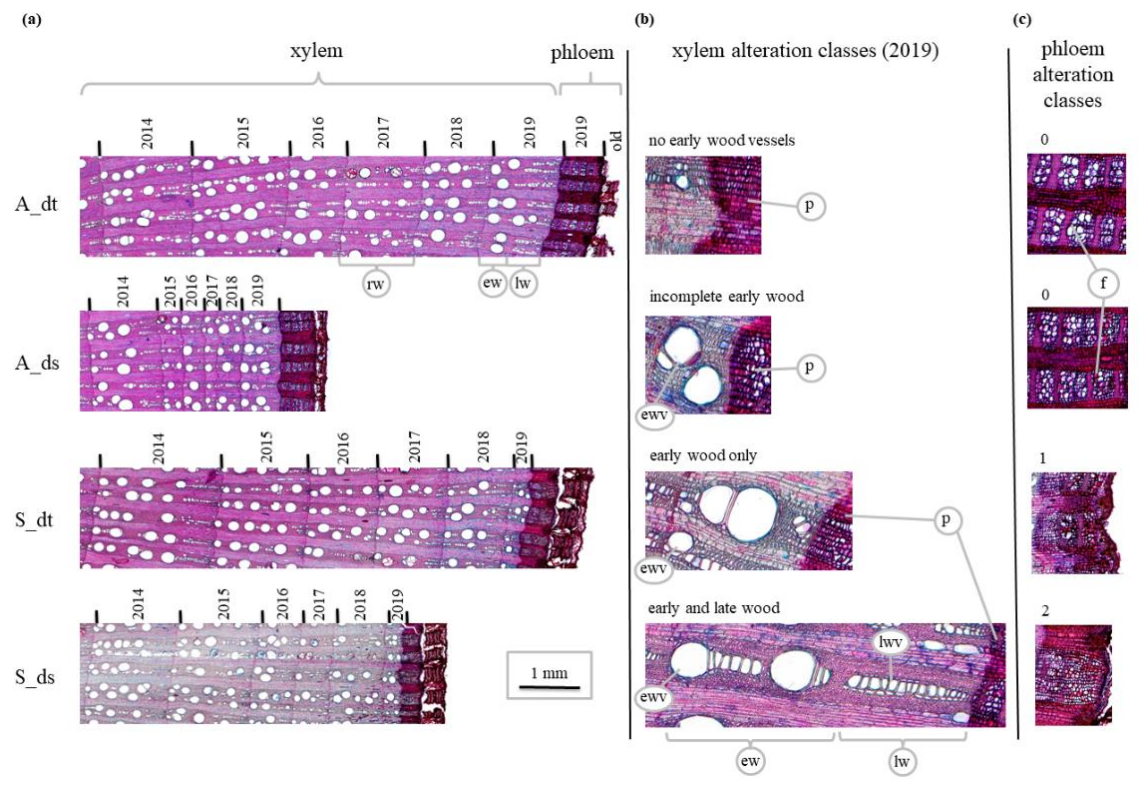
Anatomical structure of sections cored from grapevine’s trunk. (**a**) Selected 2014–2019 sections according to the four categories considered; (**b**) xylem tissue alteration classes in 2019; (**c**) phloem alteration classes according to the overall disarrangement and the presence of the fibre sclereids. A_dt = Asymptomatic and drought-tolerant; A_ds = Asymptomatic and drought-sensitive; S_dt = Symptomatic and drought-tolerant; S_ds = Symptomatic and drought-sensitive; rw = ring width; ew = early wood; lw = late wood; ewv = early wood vessel; lwv = late wood vessel; p = phloem; f = fibre.

The thin sections were visually screened for the presence of visible anatomical phloem (i.e., disruption degree of phloem fibre sclereids) and xylem modifications (i.e., portion and development of early and late wood tissue) in the year of external symptoms’ emergence (i.e., 2019). For the phloem fibre sclereids, classes representing 0%, 1–50%, and 51–100% of tissue with visible alterations related to FDp infection were assigned to each sample, as proposed by Jelmini et al. [16] (Figure 2). For the xylem alterations, the following classes were defined [28]: no early wood vessels; incomplete early wood; early wood only; early and late wood (Figure 2).

#### 2.6.2. Measurement and Standardisation of Annual Ring Widths

The width between each successive pair of tree ring limits (annual growth) was measured in two different points following the parenchyma rays with cellSens in order to calculate the mean width (Figure 2). In order to enable a direct comparison of the ring width changes within specimen groups, the obtained absolute annual ring widths of each analysed sample were then standardized at the core level by subtracting the average yearly growth of the available years between 2010 and 2018 and by dividing it by a standard deviation. The year 2019 was excluded from the calculation of the referenced average growth for standardisation because of the assumed effect of the FD symptom expression on FDp-infected specimens.

#### 2.6.3. Climatic Impact on Growth and Definition of Drought Years

In a preliminary step, possible correlations between the standardized ring width and climatic factors, such as the cumulated monthly precipitation, the mean monthly temperature, and the multiscalar climatic index SPEI (Standardized Precipitation Evapotranspiration Index) at six months-time scales (*SPEI6*; available at: https://spei.csic.es, accessed on 13 March 2022; [29]) were checked.

Since the ring width was significantly correlated only with the *SPEI6* index (*r_s_* = 0.45; *p* < 0.01; *n* = 432), the average monthly *SPEI6* value from June to September (*ySPEI6*) was then used as a proxy for the annual summer drought. The drought years were then defined as years with a *ySPEI6* value equal to or less than −1.5.

#### 2.6.4. Detection of Drought-Sensitive Specimens

The grapevine samples showing a significantly lower xylem growth (i.e., <1 Standard Deviation, SD) in the summer drought years (drought years) with respect to years without a precipitation shortage (normal years) in the period between 2010 and 2018 were classified as drought-sensitive specimens (ds). Consequently, the rest of the samples were described as drought-tolerant (dt).

#### 2.6.5. Factors Influencing Xylem Growth Responses

In order to analyse the possible impact of selected factors, such as the growing sector (different slope sectors within the vineyards), drought (drought-sensitive vs. drought-tolerant specimens), and FD symptom expression (2019 xylem growth of symptomatic and FDp-infected vs. asymptomatic FDp-free specimens), differences in ring width between different sample groups (i.e., symptomatic vs. asymptomatic) were tested for statistical significance using the non-parametric Kruskal–Wallis paired-rank test (*p* < 0.05).

Furthermore, a GLM procedure was applied in order to detect the main xylem growth drivers and their possible interactions on the absolute annual xylem ring width for all samples and for the period preceding the symptom expression (i.e., 2010–2018). As explanatory variables, the GLM included the quantitative mean annual *SPEI6* value of the 6 months-cumulated period (*ySPEI6*) and the categorical variables sample position along the slope sectors (*sector*; top, slope, or toeslope), drought sensitivity (*drought_s*: drought-sensitive vs. drought-tolerant specimens), and symptomatology (*symptom*; specimens that developed symptoms related to FDp infection (1) or that remained asymptomatic (0) in 2019, respectively). A stepwise forward procedure was applied for selecting only the factors that added explicative power and provided a significant effect to the model. The GLM analyses and Spearman’s correlations were performed and calculated using Statgraphics Centurion (StatPoint Technologies Inc., Warrenton, VA, USA). The rest of the analysis was conducted within R (version 4.1.2, R Core Team, Vienna, Austria).

## 3. Results

### 3.1. Drought Years

Table 1 reports the calculated average *SPEI6* indices from June to September and their average value of the six months-cumulated period (*ySPEI6*) for each considered year. Based on this calculation, the years 2011, 2015, 2017, and 2018, but also the year of symptomatic expression (2019), were defined as summer drought years.

Eleven specimens showed a marked (>1 SD) xylem increment reduction in the drought years and were thus classified as drought-sensitive (ds, Table 2).

### 3.2. Annual Xylem Increments and Phloem Anomalies

**Figure 3:**
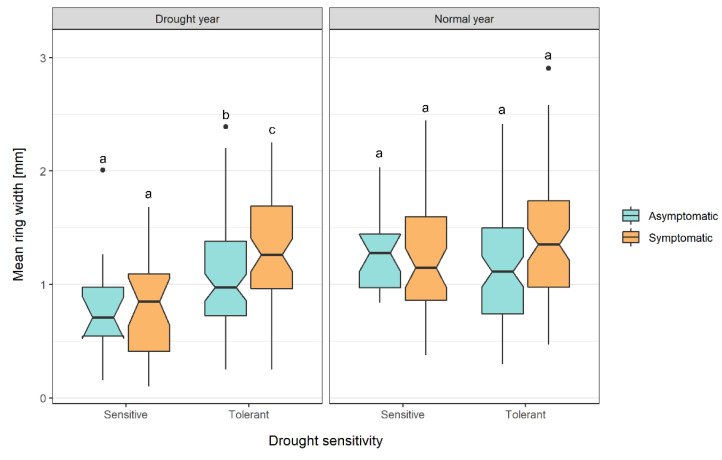
Boxplots of the mean ring width [mm] in climatic drought and normal years preceding the symptomatic expression according to drought sensitivity (drought-tolerant and drought-sensitive) and the symptomatic response of the specimens (asymptomatic and FDp-free (cyan) or symptomatic and FDp-infected (orange)). The letters indicate significant differences among the four categories per year type (Kruskal–Wallis paired test, *p* < 0.05); for the number of observations: see Table 2.

Table 2 reports the average absolute annual ring width for the four categories of the asymptomatic drought-tolerant (A_dt), asymptomatic drought-sensitive (A_ds), symptomatic drought-tolerant (S_dt), and symptomatic drought-sensitive (S_ds) in normal climatic years, in drought years, and in 2019, respectively. According to Figure 3, in the normal climatic years preceding the symptomatic expression, there are no significant differences in the absolute xylem annual width among the groups. On the contrary, in the drought years and among the drought-tolerant specimens, grapevines that were symptomatic in 2019 showed significantly higher increments with respect to the asymptomatic ones (Kruskal–Wallis paired test, *p* < 0.05). For the same year type (drought years), drought-sensitive grapevines grew significantly less than all of the other categories but without any significant difference between the grapevines that were later symptomatic or remained asymptomatic in 2019. The absolute ring width then dropped in 2019 in the symptomatic samples (S) and in the drought-sensitive ones (S_ds), in particular (Table 2).

Phloem anomalies were detected in all of the symptomatic and FDp-infected samples, while among the asymptomatic samples, only two out of twenty-six grapevines showed a slight disarrangement (Table 3). Drought sensitivity and the growing sector had no significant effect on the phloem anomalies either for the asymptomatic or for the symptomatic samples (Chi-Square test, *p* > 0.05).

During the season of external symptom expression (i.e., 2019), symptomatic specimens often formed an incomplete xylem ring lacking late wood, whereas 30.4% (seven out of twenty-three) totally failed even to produce early wood vessels (Table 2). This was not the case for the asymptomatic grapevines, most of which (84.6%, 22 out of 26) formed a full ring despite the drought conditions of the summer of 2019 (Table 2).

**Figure 4:**
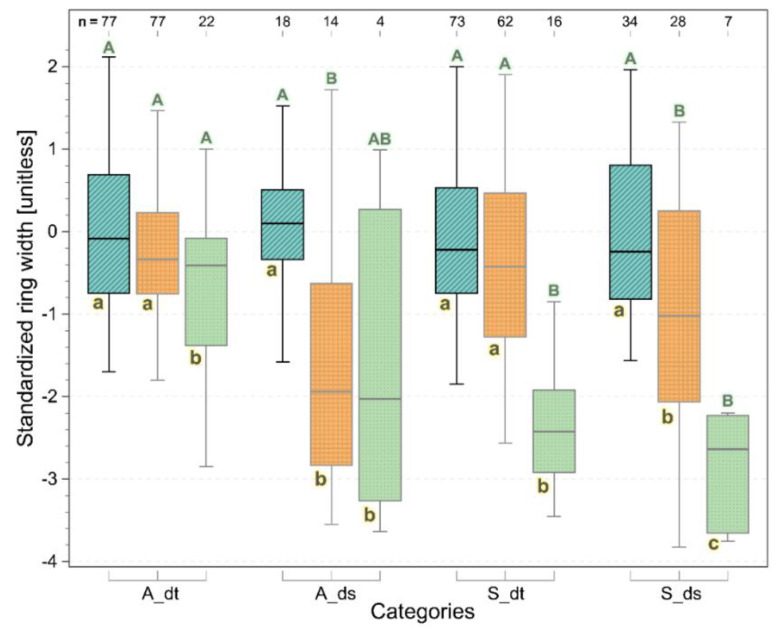
Boxplots of standardized ring width and comparison between samples grouped according to symptomatology, drought sensitivity, and year type (normal years (cyan, lines), drought years (orange, grid), and 2019 (green, dots)). A_dt (asymptomatic drought-tolerant), A_ds (asymptomatic drought-sensitive), S_dt (symptomatic drought-tolerant), and S_ds (symptomatic drought-sensitive). The drought year 2019 is considered separately because of the symptom appearance in the symptomatic and FDp-infected specimens. The letters indicate differences (Kruskal–Wallis paired-test, *p* < 0.05) between-groups (uppercase) or within-group (lowercase).

Figure 4 reports the distribution of the standardized annual xylem increments of the trunks in normal years, in drought years, and in the year of the symptom expression (i.e., 2019) of the investigated grapevines, divided between asymptomatic and symptomatic, as well as drought-tolerant and drought-sensitive specimens, respectively. Among the asymptomatic drought-tolerant individuals (A_dt), only the drought year of 2019 showed a significantly lower ring width increment, whereas, in the asymptomatic and drought-sensitive samples (A_ds), the ring width increments were significantly lower in the drought years in general (i.e., including 2019) when compared to the normal years. In the symptomatic specimens (S), the year of the symptom expression (i.e., the drought year 2019) outstands in terms of significant xylem width reductions with respect to both the drought-tolerant (S_dt) and drought-sensitive (S_ds) specimens. Moreover, the S_ds samples had significantly lower annual ring width increments also in the other drought years, i.e., not exclusively in 2019. The between-groups’ comparison highlighted the importance of drought sensitivity irrespective of the symptom appearance (i.e., 2019 vs. other drought years; see also Table 1). In fact, the drought-sensitive samples (ds) showed a significantly lower standardized ring width in the drought years than in the normal climatic years in both the asymptomatic and symptomatic samples.

**Figure 5:**
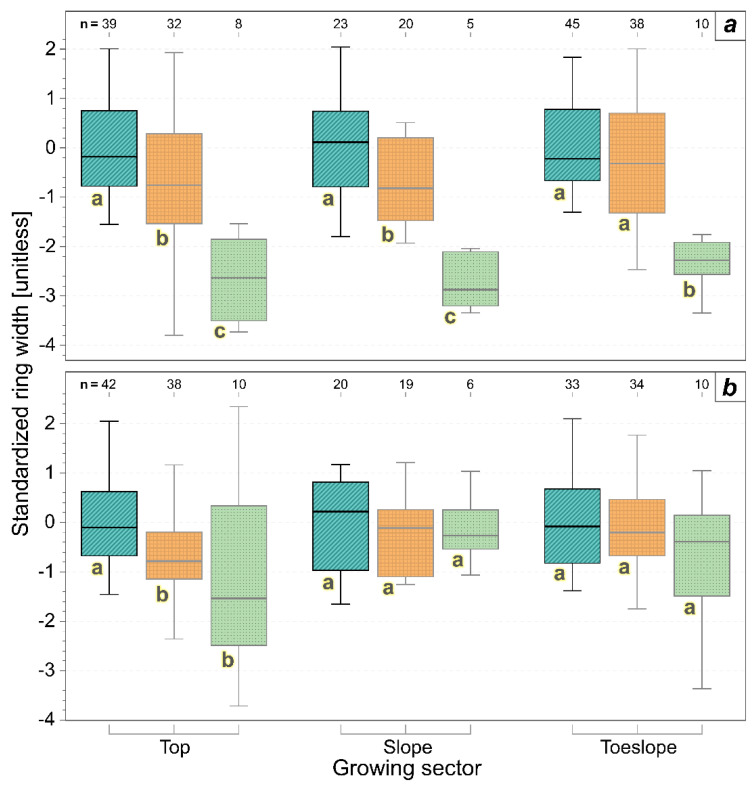
Boxplots of the standardized ring widths of symptomatic and asymptomatic samples during normal years (cyan, lines), drought years (orange, grid), and the year of symptom expression (i.e., 2019, green, dots). (**a**) symptomatic samples; (**b**) asymptomatic samples. The letters indicate significant differences (Kruskal–Wallis paired-test, *p* < 0.05) within-group for each growing sector.

When looking at the effect of the growing position with respect to the slope, only the symptomatic specimens located on the top and on the slope, and the asymptomatic ones on the top showed significant differences in ring widths between the normal and drought years, whereas the symptomatic specimens always showed significant differences in the year of symptom expressions (2019) also with respect to the other drought years (Figure 5).

### 3.3. Drivers of Xylem Increments

The GLM on the absolute ring widths fitted significantly on 383 samples in the period from 2010–2018 (*R*^2^ = 0.22, *F* = 20.72, *p* < 0.001, Table 4). According to the F-ratio, drought (i.e., *ySPEI6*, *F* = 70.76, *p* < 0.001) had the greatest influence on ring width among the factors considered, followed by the growing sector (*sector*, *F* = 9.88, *p* < 0.001), the interaction between drought sensitivity and drought (*drought_s* * *ySPEI6, F* = 7.29, *p* < 0.01), and symptomatology (*symptom*, *F* = 3.98, *p* < 0.05).

## 4. Discussion

In this paper, we used xylem ring widths and the anatomical characteristics of the trunk to analyse the response of grapevines to external stressors, such as water shortage (e.g., due to drought seasons) and the appearance of external symptoms linked to FD. As already reported by Jelmini et al. [16] for grapevine stems, also in the trunk, the phloem tissue of FDp-infected grapevines appears heavily disarranged and always accompanies a reduction in the xylem width in the coincidence of the FD symptomatic expression. In addition, and similarly to other woody plants, such as forest trees [20], the annual xylem increment in grapevine trunks reacts very sensitively towards external stressors [30]. In our specific case, the homogeneity in terms of rootstocks and scions allows us to exclude genetic-induced differences in the growth rate [31,32,33], which may, on the contrary, directly depend on environmental (i.e., the drought-induced reduction in xylem production) or disease-induced (i.e., the appearance of external FD symptoms) stresses. In this respect, pronounced drought seasons are confirmed to be the most significant driver of reductions in the xylem increments, as already shown by Munitz et al. [30], and are highlighted by the overall high relevance of the climatic signal (i.e., SPEI index) in the GLM outputs. The synergetic effect of an intrinsic sensitivity to drought of the concerned grapevines further exacerbates this reduction, as attested by the significant interaction of the drought season with the drought sensitivity of the grapevines. Moreover, the assumed water availability gradient due to the slope geomorphology (i.e., an increasing water availability from the top to the toeslope) was also reflected in the results, although with a lower effect than climatic conditions. This is probably due to the lack of a sharp geomorphological gradient within the considered vineyard and/or the plastic ability of the grapevines to access and manage water even in harsher conditions [34,35].

The emergence of external symptoms linked to FD in the drought season of 2019 revealed to be an additional and very clear reduction factor of the annual xylem width, irrespectively of the drought sensitivity of the concerned individuals. In this respect, asymptomatic drought-sensitive specimens (A_ds) only showed trends and no significant differences when compared to the symptomatic samples (S), probably because of the low sample number (only four) and the related high sample variability (Figure 4). Anyway, this suggests that FDp infections supersede the effect of drought in the indirect reduction of xylem growth. Interestingly, among the drought-tolerant specimens, the symptomatic individuals showed a significantly higher growing performance with respect to the asymptomatic ones. Considering that the years since 2017 are characterized as drought years (see the *ySPEI6* values in Table 1), we can assume that the infection of the symptomatic specimens took place in a drought year and, alas, during a higher growing performance for the drought-tolerant specimens. This may suggest existing growth-related (and, thus, probably also vitality-related) feeding preferences of the main FDp vector, *S. titanus*, as already hypothesised by several authors (e.g., [36,37,38]).

The reductions in the xylem increments in the year of the symptom expression resulted, in some cases, in an incomplete formation of the early wood tissue, letting us assume that the alteration of the plant physiology starts early in the season or just after the early wood development, which, in grapevines, usually takes place before the end of June [30,39]. Possible or partial anatomical responses before the external symptom expression have already been reported by Jelmini et al. [16] for the asymptomatic stems of FDp-infected grapevines of the susceptible cultivar ‘Chardonnay.’ Such inhibition of the xylem tissue formation during the season of the FD symptom expression supports the possible infection after inoculation by *S. titanus* in the previous year, followed by the overwintering of FDp inside the plant and the expression of symptoms in the following vegetative season [11]. From a methodological point of view, although annual leaf tissues are conducive to the diagnosis of the disease in its acute phase, the search for FDp-infected specimens for research purposes could be extended to permanent organs, such as the fine roots where other GY phytoplasmas, such as ‘*Candidatus* Phytoplasma solani,’ which causes “Bois noir,” are known to persist for at least five years after the disappearance of the last leaf symptoms of the recovered plants [40]. The limitations of foliar diagnosis were also highlighted by Morone et al. [41] when they tested the leaf tissues of the asymptomatic stems of FDp-infected grapevines, which usually resulted in being FDp-free. Margaria et al. [42] and Pacifico et al. [43] also came to similar conclusions when testing leaf tissues sampled from recovered grapevines without external symptom expression. The approach presented in this work, thus, highlights the potential for developing early diagnostic protocols for research applications under controlled conditions capable of detecting the presence of phytoplasma in plant organs, such as roots, where phytoplasmas could take refuge during the latent overwintering phase or after recovery of the plant.

## 5. Conclusions

Even though FDp does not inhabit and replicate inside the xylem tissue, the present work, along with that of Jelmini et al. [16], shows that there is an indirect inhibition of ring growth and xylem tissue formation in FDp-infected grapevines. Moreover, the stress-induced effect of drought in terms of xylem growth seems to be superseded by the infection-induced stress when grapevines express external FD symptoms. The applied dendroecological approach showed to be a good method for the quantification of xylem growth impairments in grapevines infected by phytoplasmas, but other approaches are needed in order to further investigate the actual mechanisms that lead to significant wood growth reductions in FDp-infected grapevines.

## Figures and Tables

**Table 1:** Values of *SPEI6* (Standardized Precipitation Evapotranspiration Index over six months-time scale) for each summertime month and the average of the JJAS-SPEI value of the six months-cumulated period (*ySPEI6*) within the period 2010–2019. Drought years are reported in bold. See Vicente-Serrano et al. [29] for further details on SPEI.

Year	June	July	August	September	*ySPEI6*
2010	−0.450	−0.407	0.272	0.059	−0.131
**2011**	**−2.181**	**−1.494**	**−1.543**	**−1.545**	**−** **1.691**
2012	−0.688	−1.137	−0.684	−0.254	−0.692
2013	0.378	0.336	0.083	0.227	0.256
2014	−0.969	−0.757	−1.163	−1.523	−1.103
**2015**	**−1.134**	**−1.849**	**−2.048**	**−1.881**	**−** **1.728**
2016	0.092	−0.315	−1.215	−1.494	−0.733
**2017**	**−1.477**	**−1.700**	**−1.824**	**−1.990**	**−** **1.748**
**2018**	**−1.297**	**−2.309**	**−2.580**	**−2.836**	**−** **2.255**
**2019**	**−1.638**	**−1.957**	**−1.937**	**−2.047**	**−** **1.895**

**Table 2:** Xylem anatomical features in 2019 and average annual ring width according to the four main categories of asymptomatic drought-tolerant (A_dt), asymptomatic drought-sensitive (A_ds), symptomatic drought-tolerant (S_dt), and symptomatic drought-sensitive (S_ds) in normal climatic years, in drought years, and in 2019. Number of observed and analysed rings per year type: N. Obs; standard deviation: SD.

Category	Number of Specimens	Xylem Anatomical Features 2019				Xylem Annual Widths			
		No Vessels	Incomplete Early Wood	Early Wood Only	Early and Late Wood	Year Type	Annual Rings		
							N. Obs	Average Width (mm)	SD (mm)
A_dt	22	0	3	0	19	normal *	79	1.202	0.24
						drought ^†^	77	1.123	0.38
						2019	22	1.020	0.45
A_ds	4	0	1	0	3	normal *	21	1.305	0.23
						drought ^†^	14	0.776	0.07
						2019	4	0.751	0.52
S_dt	16	3	1	12	0	normal *	77	1.443	0.27
						drought ^†^	62	1.277	0.23
						2019	16	0.349	0.18
S_ds	7	4	0	3	0	normal *	45	1.261	0.30
						drought ^†^	28	0.783	0.28
						2019	7	0.164	0.09

* years 2010, 2012, 2013, 2014, and 2016. ^†^ years 2011, 2015, 2017, and 2018.

**Table 3:** Number of samples with phloem anomalies according to the four main categories of asymptomatic drought-tolerant (A_dt), asymptomatic drought-sensitive (A_ds), symptomatic drought-tolerant (S_dt), and symptomatic drought-sensitive (S_ds) and the growing sector along the slope (top, slope, and toeslope). N: number of analysed samples per category and sector.

Category	Growing Sector	N	Samples with Phloem Anomalies in 2019		
			0%	1–50%	51–100%
A_dt	top	8	7	1	0
	slope	5	5	0	0
	toeslope	9	9	0	0
	total	22	21	1	0
A_ds	top	2	2	0	0
	slope	1	1	0	0
	toeslope	1	0	1	0
	total	4	3	1	0
S_dt	top	3	0	1	2
	slope	4	0	0	4
	toeslope	9	0	3	6
	total	16	0	4	12
S_ds	top	5	0	0	5
	slope	1	0	1	0
	toeslope	1	0	0	1
	total	7	0	1	6

**Table 4:** Generalized linear model (GLM) for the absolute ring widths in the period from 2010–2018.

GLM Statistics				Predictor	df	*F*	*p*
Response	*R* ^2^	*F*	*p*				
Ring width	0.22	20.72	< 0.001	*symptom*	1, 382	3.98	<0.05
				*sector*	2, 382	9.88	<0.001
				*ySPEI_6*	1, 382	70.76	<0.001
				*drought_s* * *ySPEI_6*	1, 382	7.29	<0.01

Predictors: *symptom* = specimens with and without symptom expression in 2019; *sector* = growing site with respect to the slope sectors (top, slope, and toeslope); *ySPEI6* = annual mean value of monthly standardized precipitation evapotranspiration index at six months-time scale; *drought_s* = drought sensitivity; drought_s * ySPEI6 = interaction between *drought_s* and *ySPEI6*.

## Data Availability

Data supporting the reported results can be found at https://doi.org/10.16904/envidat.324.
